# Recurrent Middle Eastern Differentiated Thyroid Carcinoma Has Worse Outcomes Than Persistent Disease

**DOI:** 10.3390/jcm13071877

**Published:** 2024-03-25

**Authors:** Sandeep Kumar Parvathareddy, Abdul K. Siraj, Padmanaban Annaiyappanaidu, Saeeda O. Ahmed, Saif S. Al-Sobhi, Fouad Al-Dayel, Khawla S. Al-Kuraya

**Affiliations:** 1 Human Cancer Genomic Research, Research Centre King Faisal Specialist Hospital and Research Centre, P.O. Box 3354, Riyadh 11211, Saudi Arabia; psandeepkumar@kfshrc.edu.sa (S.K.P.); asiraj@kfshrc.edu.sa (A.K.S.); pannaiyappanaidu97@kfshrc.edu.sa (P.A.); ahmsaeeda@kfshrc.edu.sa (S.O.A.); 2 Department of Surgery, King Faisal Specialist Hospital and Research Centre, P.O. Box 3354, Riyadh 11211, Saudi Arabia; sobhi@kfshrc.edu.sa; 3 Department of Pathology, King Faisal Specialist Hospital and Research Centre, P.O. Box 3354, Riyadh 11211, Saudi Arabia; dayelf@kfshrc.edu.sa

**Keywords:** differentiated thyroid carcinoma, recurrence, persistence, overall survival, cancer-specific survival

## Abstract

**Background:** Despite the excellent prognosis of differentiated thyroid carcinoma (DTC), recurrent and persistent disease remain major challenges. Emerging studies to differentiate between recurrent and persistent disease are controversial, with studies from the Middle East lacking. **Methods:** We retrospectively analyzed 1691 patients who underwent surgery ± I131 treatment for DTC, with a median age of 38.7 years and median follow-up of 95.3 months. **Results:** We found a similar prevalence rate for persistent and recurrent disease (17.7% vs. 17.9%) in Middle Eastern DTC patients. Relative to patients with persistent disease, patients with recurrent disease were significantly older (median age: 36.1 vs. 45.8 years; *p* < 0.0001) and were more likely to have ATA high-risk tumors (61.5% vs. 75.2%; *p* = 0.0003). On multivariate logistic regression analysis, both T and N status were independent predictors for recurrent as well as structural persistent disease. However, older age, bilaterality and extrathyroidal extension were independent predictors of recurrent disease alone. In addition, patients with recurrent disease had significantly worse cancer-specific survival (*p* < 0.0001), which remained significant in multivariate analysis. **Conclusions:** Although persistent and recurrent disease in Middle Eastern DTC have similar frequencies, recurrent disease has worse outcomes compared to persistent disease. Hence, differentiating recurrence from persistence has great potential clinical relevance for therapeutic and follow-up approaches, contributing to improving the outcomes of DTC patients of Middle Eastern ethnicity.

## 1. Introduction

Differentiated thyroid carcinoma (DTC) is the commonest endocrine malignancy and generally associated with excellent survival [1,2,3]. Adequate initial management, including surgery, in some cases followed by radioactive iodine (RAI) and thyrotropin suppression therapy, leads to a high likelihood of cure in these patients [4,5,6,7]. Despite the high cure rate, disease recurrence and persistence remain major challenges for patients and physicians [8,9].

Most published studies have considered persistent and recurrent DTC to form a single entity [10,11,12]. The definition and timeline of recurrence and persistence is universal in the literature. According to the recent ATA guidelines, disease-free status is defined as no clinical evidence of a tumor, no evidence of a tumor based on RAI imaging or ultrasound, unstimulated Tg < 0.2 mg/mL or stimulated Tg < 1 mg/mL in the absence of antibodies. However, from a clinical perspective, the disease is considered persistent when detected a short time after initial therapy and recurrent when detected after a disease-free period of ≥one year [4]. Although a few studies have tried to differentiate between persistent and recurrent disease [13,14], they could not identify any differences between these two conditions with regard to patient prognosis. However, one recent study involving a large series of DTC patients of European ethnicity has demonstrated a significant difference between persistent and recurrent disease in terms of outcomes and clinical implications [15].

Previously, we investigated the recurrence rate in Middle Eastern papillary thyroid carcinoma (PTC) [16]. However, we did not investigate the difference between recurrence and persistence in DTC in the Middle East, where PTC is considered the second commonest cancer affecting females [17]. Most studies have considered recurrence and persistence to be a single entity or used them interchangeably. Their impact on management and outcomes for these two entities separately has not been explored thoroughly. There is ongoing debate about the necessity to differentiate between persistent and recurrent disease, and the results have been inconsistent. Moreover, there is a paucity of data regarding recurrence and persistence in patients of this ethnicity. A better understanding of these entities could result in better patient management and surveillance.

The goal of this study was to determine the frequency of recurrent and persistent diseases, their clinico-pathological associations and, most importantly, the difference in outcomes (cancer-specific survival) between recurrent and persistent disease in DTC, as well as its clinical implications. To achieve this, we retrospectively evaluated a series of 1691 Middle Eastern patients with DTC.

## 2. Materials and Methods

### 2.1. Patient Selection and Clinico-Pathological Data

One thousand eight-hundred and twenty-two consecutive DTC patients diagnosed between 1988 and 2020 at King Faisal Specialist Hospital and Research Centre (Riyadh, Saudi Arabia) were eligible to be included in this study. Patients of Middle Eastern origin were included in the study, with patients of other nationalities/ethnicities excluded. Also, patients with follow-up durations of less than 5 years who had not developed recurrent disease were excluded. Finally, 1691 patients were included for analysis. Cases were identified based on clinical history followed by fine-needle aspiration cytology for confirmation. The Institutional Review Board of the hospital approved this study, and since only retrospective patient data were utilized, the Research Advisory Council (RAC) provided a waiver of consent under project RAC # 221 1168 and # 2110 031.

All patients underwent surgery (81.5% underwent total thyroidectomy and 18.5% underwent hemithyroidectomy/lobectomy), performed by board-certified, highly experienced endocrine surgeons who have performed high volumes of thyroidectomies (>25 thyroidectomies/year). Given that the patients included in our study had been diagnosed between 1988 to 2020, the management protocols varied based on the changes in international standards over time. However, our institute has been following the recommendations of the American Thyroid Association (ATA) guidelines since they were first published in 1996 and modified the protocols based on subsequent revisions of the ATA guidelines.

Overall, 77.9% (1317/1691) of patients received RAI therapy. Patients were seen 6 to 8 weeks following surgery, having been prepared with thyroid hormone withdrawal for at least four weeks and a low-iodine diet for one week. The ATA guidelines [4] were used to determine the RAI dosages administered. Previously, the administered dosage used to be 100 mCi for remnant ablation, 100–150 mCi if there are lymph node metastases and 150–200 mCi if there are distant metastases. However, in the last 5 years, a trend towards more conservative doses of RAI was adopted, with most patients receiving 30–100 mCi for thyroid remnant ablation and 100–200 mCi for patients with lymph node or distant metastases. In addition, for pediatric patients, the dosage was adjusted to the child’s body weight (based on adult dosage of 70 kg person). 

Baseline clinico-pathological data were collected from case records and are summarized in Table 1. Staging of DTC was performed using the eighth edition of American Joint Committee on Cancer (AJCC) staging system [18]. 

### 2.2. Definitions of Persistent and Recurrent Disease

All patients were followed up at regular intervals after surgery (±RAI therapy) with neck ultrasound, CT scan, post-therapy 131I WBS (for patients who underwent RAI therapy), a thyroid function test and serum Tg and anti-Tg antibody measurements (Tg and anti-Tg were analyzed only for patients who underwent total thyroidectomy). 

Patients were grouped according to disease status, with patients considered as having an excellent response if there was an absence of clinical, biochemical (unstimulated serum thyroglobulin (Tg) levels of <0.2 µg/L or stimulated Tg levels of <1 µg/L in the absence of interfering thyroglobulin antibodies (TgAb)) or structural disease (locoregional lymph node metastasis as evidenced by neck ultrasound and pathologically confirmed by fine needle aspiration biopsy or the appearance of distant metastases in the bone, lung or brain as evidenced by 131I WBS or CT scan). In contrast, active disease was defined by the presence of biochemical (unstimulated serum Tg levels ≥ 0.2 µg/L or stimulated Tg levels ≥ 1 µg/L; the rising or de novo appearance of TgAb) or structural (abnormal findings on radio-imaging) disease. Presence of active disease within 12 months post-operatively was defined as persistent disease, whereas patients who had excellent response for at least 12 months post-operatively and later developed active disease were defined as having recurrent disease, based on ATA guidelines and previous publications [4,13,15].

### 2.3. Study End-Point

The study end-point was marked by an analysis of the difference in outcomes (cancer-specific survival (CSS)) between recurrent and persistent disease in DTC. CSS was defined as the time from diagnosis to death due to DTC progression.

### 2.4. Statistical Analysis

The associations between clinico-pathological variables and recurrence/persistence were determined using contingency table analysis and Chi square tests. A Mantel–Cox log-rank test was used to evaluate CSS. Survival curves were generated using the Kaplan–Meier method. Logistic regression analysis and a Cox proportional hazards model was used for univariate and multivariate analysis. Two-sided tests were used for statistical analyses, with the limit of significance defined as a *p* value < 0.05. Data analyses were performed using the JMP14.0 (SAS Institute, Inc., Cary, NC, USA) software package.

## 3. Results

### 3.1. Patient and Tumor Characteristics

The median age of the study population was 38.7 years (inter-quartile range: 29.1–51.0 years), with a male-to-female ratio of 1:3. The majority of tumors were PTC (94.2%; 1593/1691). A total of 31.4% (529/1691) of tumors were bilateral and 48.2% (811/1691) were multifocal. A total of 40.4% (684/1691) of tumors exhibited extrathyroidal extension and 27.9% (471/1691) showed lymphovascular invasion. Lymph node metastasis was noted in 47.4% (802/1691) and distant metastasis at diagnosis in 5.4% (92/1691) of DTCs (Table 1).

The median follow-up was 95.3 months (range 12–362 months). In the entire cohort, 3.0% (51/1691) of patients died, with cancer-specific deaths accounting for 2.1% (36/1691) of cases. The corresponding incidence for recurrent disease was 9.2% (28/303) overall deaths and 8.9% (27/303) for cancer-specific deaths, whereas for persistent disease, it was 2.3% (7/299) overall deaths and 1.3% (4/299) for cancer-specific deaths.

### 3.2. Incidence and Treatment of Persistent and Recurrent Disease

The majority of the patients with DTC (64.4%; 1089/1691) had an excellent response after initial surgery ± RAI therapy. Persistent disease was noted in 17.7% of patients (299/1691), with biochemical persistence accounting for 27.8% (83/299) and structural persistence for 72.2% (216/299) of these cases. Recurrent disease was seen in 17.9% (303/1691) of patients in our cohort, with biochemical recurrence accounting for 26.7% (81/303) and structural persistence for 73.3% (222/303) of these cases (Table 2).

Supplementary Figures S1 and S2 show the initial treatments performed, the outcome after the initial treatment (in terms of recurrence and persistence after 12 months) and additional treatments that were performed after recurrent/persistent disease.

### 3.3. Clinico-Pathological Associations and Predictors for Persistent and Recurrent DTC

Persistent disease was significantly associated with bilateral tumors (*p* < 0.0001), multifocality (*p* = 0.0002), extrathyroidal extension (*p* < 0.0001), advanced T stage (*p* < 0.0001), lymph node metastasis (*p* < 0.0001), stage IV tumors (*p* < 0.0001), higher cumulative RAI dosage (*p* < 0.0001) and a higher number of RAI doses (*p* < 0.0001). Recurrent disease was found to be associated with older age (*p* < 0.0001), male gender (*p* = 0.0003), bilateral tumors (*p* < 0.0001), multifocality (*p* = 0.0045), extrathyroidal extension (*p* < 0.0001), advanced T stage (*p* > 0.0001), lymph node metastasis (*p* < 0.0001), stage IV tumors (*p* < 0.0001), higher cumulative RAI dosage (*p* < 0.0001) and a higher number of RAI doses (*p* < 0.0001) (Table 3). ATA high risk was associated with both persistent and recurrent disease, but the incidence was significantly higher in recurrent disease compared to persistent disease (75.2% vs. 61.5%, *p* = 0.0003). 

On multivariate analysis, we found that T status (T3 and T4 tumors; OR = 3.50; 95% CI = 2.37–5.17; *p* < 0.0001 and OR = 6.95; 95% CI = 3.59–13.45; *p* < 0.0001, respectively) and N status (N1a and N1b; OR = 7.51; 95% CI = 4.75–11.86; *p* < 0.0001 and OR = 9.37; 95% CI = 6.26–14.01; *p* < 0.0001, respectively) were significant independent predictors for persistent disease. Older age (OR = 2.43; 95% CI = 1.70–3.46; *p* < 0.0001), bilateral tumors (OR = 2.30; 95% CI = 1.41–3.75; *p* = 0.0008), extrathyroidal extension (OR = 2.30; 95% CI = 1.67–3.16; *p* < 0.0001), T status (T4 tumors; OR = 4.31; 95% CI = 2.36–7.86; *p* < 0.0001) and N status (N1a and N1b; OR = 2.47; 95% CI = 1.54–3.97; *p* = 0.0002 and OR = 4.65; 95% CI = 3.26–6.63; *p* < 0.0001) were found to be independent predictors for recurrent disease (Table 4). 

### 3.4. Survival Outcomes for Persistent and Recurrent Disease

**Figure 1:**
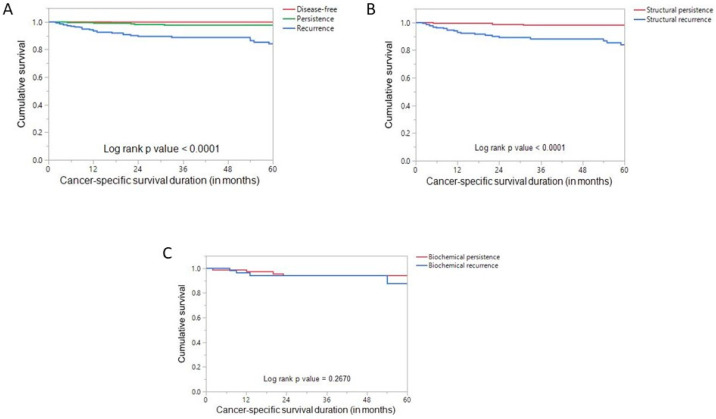
Cancer-specific survival. (**A**) Kaplan–Meier survival curve showing poor cancer-specific survival in patients with recurrent disease compared to those with persistent disease. (**B**) Kaplan–Meier survival curve showing poor cancer-specific survival in patients with structural recurrent disease compared to those with structural persistent disease. (**C**) Kaplan–Meier survival curve showing no significant difference in cancer-specific survival in patients with biochemical recurrent disease compared to those with biochemical persistent disease.

Patients with recurrent disease had a significantly worse CSS (*p* < 0.0001) compared to those with persistent disease (Figure 1A). We further divided the persistent and recurrent disease patients into structural and biochemical subgroups given the different risk profiles and treatment regimens for these two subgroups. Patients with structural recurrent disease had a significantly worse CSS (*p* < 0.0001) compared to those with structural persistent disease (Figure 1B). On multivariate Cox proportional hazards analysis, recurrent disease was found to be an independent predictor of worse CSS (hazard ratio = 6.44; 95% confidence interval = 2.40–22.56; *p* < 0.0001) (Table 5).

However, the difference in CSS was not significant between patients with biochemical recurrent and biochemical persistent disease (*p* = 0.2670) (Figure 1C).

### 3.5. Long-Term Outcomes for DTC Patients with Persistent Disease

Of the 299 patients with persistent disease, 161 (53.8%) patients underwent additional surgery alone and 22 (7.4%) had additional surgery and RAI therapy, whereas 95 (31.8%) patients received additional doses of RAI therapy alone (ranging from two to seven). Twenty-one patients did not receive any additional therapies (Supplementary Figure S2). Following additional therapies, 142 (48.8%) of the 299 patients with persistent disease were free of disease. At long-term follow-up, 16 of these patients had a relapse and 126 patients remained disease-free at the last follow-up (Table 2).

## 4. Discussion

In this study, we retrospectively analyzed a large cohort of 1691 DTC patients of Middle Eastern ethnicity. Overall, recurrence after initial treatment is relatively common and has been reported to vary between 3% and 23% [19,20,21,22], whereas persistent disease has been reported in in up to 23% of DTCs [15,23,24]. We found that 35.6% (602/1691) of DTC patients had disease events (persistence or recurrence) after initial therapy. This had a huge impact on the patients’ subsequent therapy and follow-up.

With a relatively high rate of disease events, we ended up with a large investigated cohort of 602 cases with disease after initial therapy, which adds strength to our findings. Interestingly, in this cohort, we found a nearly equal frequency of recurrent (50.3%) and persistent disease (49.7%). Most of the previous studies [10,11,12] have evaluated and considered recurrent and persistent disease as a single entity despite the differentiation being made recently by the ATA guidelines, where clear definitions of both conditions have been clarified [4]. However, the distinction between persistent and recurrent disease is still controversial in the literature [13,14,15] and is lacking in DTCs from Saudi Arabia, where PTC (the commonest type of DTC) ranks as the second most common cancer affecting Saudi females, after breast cancer. 

Although no significant difference in incidence between recurrent and persistent disease (16.6% vs. 16.4%) was noted, we found that T3/T4 status and cervical lymph node involvement (N1a/b) were likely to predict persistent DTC. Lymph node metastasis was also a predictor of recurrent disease. However, older age, bilateral tumors and extrathyroidal extension were independent predictors of recurrent disease, but not persistent disease. These variables have been shown to increase the risk of recurrence in previous studies [13,25,26,27].

In this study, DTC recurrence and persistence were, in fact, considered two separate clinical conditions in terms of patient prognosis and outcomes. Despite equal frequencies of both conditions, patients with recurrent disease clearly had worse CSS, which remained significant on multivariate analysis. The worse outcome of recurrent disease is most likely the consequence of more aggressive clinical associations at presentation. Furthermore, the incidence of ATA high-risk tumors in our cohort was relatively high. This could partially be attributed to the fact that, as a tertiary care institute, patients with advanced disease from all over Saudi Arabia are referred to our hospital. In addition, previous studies from the Middle East have shown a higher incidence of advanced disease [28,29], suggesting that either DTC in this population is inherently more aggressive or there is a delay in patients seeking medical help (or a combination of both). Additionally, rescue therapies with newer agents, such as Lenvatinib and Sorafenib, have only been used for patients with tumor recurrence at our institute since 2019. However, considering that the patients included in our cohort were diagnosed between 1988 and 2020, the majority of patients with recurrent tumors have not received these newer therapies. This could be another reason for the relatively high mortality rate in patients with tumor recurrence in our cohort. Indeed, on further classification, the worst outcome was limited to patients with structural recurrence, not those with biochemical recurrence. This has important clinical implications given the different risk profiles and treatment regimens for these two subgroups.

A previous large study [15] using the same clinical criteria for defining recurrence and persistence, conducted on a European cohort, was able to demonstrate differences in clinical outcomes between recurrent and persistent DTC. However, our results are opposite to their results, as they showed that persistent disease had worse outcomes than recurrent DTC. These contrasting results could be attributed to ethnic differences and might suggest differences in the severity of DTC at the time of presentation. Previous reports have also highlighted the role of ethnicity in the patient characteristics and clinical presentation of DTC among different ethnic groups [30,31,32]. The association of metastasis with recurrence in Middle Eastern DTC may be indicative of a greater extent of disease at the time of presentation, placing patients at higher risk of true recurrence and worse outcomes. Indeed, a higher percentage of deaths was noted in patients with recurrent disease in our cohort due to the development of distant metastasis and progression of disease. Of the 303 patients with recurrent disease, 11.2% (34/303) died overall, and 32 of these 34 patients died due to development of distant metastasis, whereas of the 299 patients with persistent disease, 2.3% (7/299) died overall, and 3 of these 7 patients died due to development of distant metastasis.

One of the limitations of this study is that it is retrospective and from a single tertiary care institute, which could give rise to selection bias. Also, the findings of this study were obtained from a very specific ethnicity, and therefore, generalization of the conclusions should be undertaken with caution. Additionally, although the surgeries were performed by board-certified, highly experienced endocrine surgeons who have performed high volumes of thyroidectomies, we do acknowledge that some patients might have had inadequate initial treatment, leading to increased incidence of recurrent/persistent disease.

## 5. Conclusions

This study showed that Middle Eastern patients with DTC after initial therapy had similar frequencies of persistent and recurrent disease. However, differences in predictive risk factors in patients with recurrent and persistent disease were observed. Recurrent disease was found to be associated with more aggressive clinico-pathological characteristics and was a strong predictor of worse cancer-specific survival compared to persistent disease in our cohort of Middle Eastern DTC patients. Our findings will help physicians and surgeons in understanding the difference between persistent and recurrent disease and ensure adequate initial surgery and long-term follow-up in patients at risk for recurrent disease.

## Figures and Tables

**Table 1:** Clinico-pathological characteristics of differentiated thyroid carcinoma patient cohort.

Clinico-Pathological Variables	*n*	%
Total	1691	
Age (years)		
Median (IQR), years	38.7 (29.1–51.0)	
<55	1369	81.0
≥55	322	19.0
Gender		
Female	1279	75.6
Male	412	24.4
Histologic subtype		
Papillary thyroid carcinoma	1593	94.2
Follicular thyroid carcinoma	98	5.8
Tumor laterality		
Unilateral	1154	68.6
Bilateral	529	31.4
Multifocality		
Yes	811	48.2
No	872	51.8
Extrathyroidal extension		
Present	684	40.4
Absent	1007	59.6
Lymphovascular invasion		
Present	471	27.9
Absent	1220	72.1
pT		
T1	658	39.0
T2	539	31.9
T3	369	21.9
T4	121	7.2
pN		
N0	680	40.2
Nx	209	12.4
N1a	237	14.0
N1b	565	33.4
pM		
M0	1599	94.6
M1	92	5.4
TNM stage		
I	1419	84.3
II	183	10.9
III	21	1.2
IV	61	3.6
Disease status		
Disease-free	1089	64.4
Persistent disease	299	17.7
Recurrent disease	303	17.9
ATA risk category		
Low	318	18.8
Intermediate	579	34.2
High	794	47.0
Follow-up duration, median (IQR), months	95.3 (62.0–144.6)	

IQR—inter-quartile range.

**Table 2:** Incidence of persistent and recurrent disease in patients with DTC.

Characteristic	Persistent Disease, *n* (%)	Recurrent Disease, *n* (%)
Incidence	299 (17.7)	303 (17.9)
Biochemical	83/299 (27.8)	81/303 (26.7)
Structural	216/299 (72.2)	222/303 (73.3)
Only lymph node metastasis	175/299 (58.5)	120/303 (39.6)
Central	108/299 (36.1%)	77/303 (25.4%)
Lateral	67/299 (22.4%)	43/303 (14.2%)
Only distant metastasis	30/299 (10.0)	83/303 (27.4)
Both	11/299 (3.7)	19/303 (6.3)
Disease-free at last visit	126/299 (42.2)	97/303 (32.0)

**Table 3:** Clinico-pathological associations of persistent and recurrent disease compared to excellent response in patients with differentiated thyroid carcinoma.

	Excellent Response		Persistence		*p* Value	Recurrence		*p* Value
	No.	%	No.	%		No.	%	
Total	1089	64.4	299	17.7		303	17.9	
Follow-up duration, median (IQR), months	87.6 (62.4–129.6)		102 (68.4–140.4)			111.6 (60–181.2)		
Age (years)								
Median (IQR), years	38.0 (29.6–49)		36.1 (27.4–48.8)		0.3940	45.8 (30–60)		<0.0001
<55	917	84.2	248	82.9	0.6006	204	67.3	<0.0001
≥55	172	15.8	51	17.1		99	32.7	
Gender								
Female	850	78.0	224	74.9	0.2550	205	67.7	0.0003
Male	239	22.0	75	25.1		98	32.3	
Histologic subtype								
Papillary	1003	92.1	297	99.3	<0.0001	293	96.7	0.0026
Follicular	86	7.9	2	0.7		10	3.3	
Tumor laterality								
Unilateral	803	74.1	181	60.7	<0.0001	170	56.3	<0.0001
Bilateral	280	25.9	117	39.3		132	43.7	
Multifocality								
Yes	481	44.4	168	56.4	0.0002	162	53.6	0.0045
No	602	55.6	130	43.6		140	46.4	
Extrathyroidal extension								
Present	309	28.4	176	58.9	<0.0001	199	65.6	<0.0001
Absent	780	71.6	123	41.1		104	34.3	
Lymphovascular invasion								
Present	306	28.1	88	29.4	0.6517	77	25.4	0.3515
Absent	783	71.9	211	10.6		226	74.6	
pT								
T1	472	43.4	75	25.2	<0.0001	111	36.8	<0.0001
T2	396	36.4	74	24.8		69	22.8	
T3	195	17.9	106	35.6		68	22.5	
T4	24	2.2	43	14.4		54	17.9	
pN								
N0	563	51.7	42	14.1	<0.0001	72	23.8	<0.0001
Nx	183	16.8	9	3.0		8	2.6	
N1a	124	11.4	74	24.7		39	12.9	
N1b	219	20.1	174	58.2		172	56.8	
TNM Stage								
I	981	90.6	242	81.2	<0.0001	196	64.7	<0.0001
II	80	7.4	32	10.7		71	23.4	
III	7	0.6	8	2.7		6	2.0	
IV	15	1.4	16	5.4		30	9.9	
ATA risk category								
Low	288	26.4	15	5.0	<0.0001	15	5.0	<0.0001
Intermediate	419	38.5	100	33.4		60	19.8	
High	382	35.1	184	61.5		228	75.2	
Cumulative RAI dosage (mCi)								
Mean (±S.D.)	130.2 ± 75.6		179.8 ± 120.4		<0.0001	276.8 ± 192.1		<0.0001
Median (range)	120 (29–720)		150 (28–931)			200 (54–1160)		
Number of RAI doses								
Mean (±S.D.)	1.1 ± 0.4		1.3 ± 0.7		<0.0001	1.8 ± 1.0		<0.0001
Median (range)	1.0 (0–7.0)		1.0 (0–7.0)			1.0 (0–7.0)		

IQR—inter-quartile range, ATA—American Thyroid Association, RAI—radioactive iodine, mCi—millicurie, S.D.—standard deviation.

**Table 4:** Multivariate analysis of risk factors for predicting persistent and recurrent disease.

	Persistent Disease		Recurrent Disease	
Risk Factor	OR (95% CI)	*p* Value	OR (95% CI)	*p* Value
Male sex	Not significant at univariate		1.28 (0.92–1.78)	0.1432
Age (>55 years)	Not significant at univariate		2.43 (1.70–3.46)	<0.0001
Bilateral tumors	1.17 (0.75–1.81)	0.4876	2.30 (1.41–3.75)	0.0008
Multifocal tumors	0.99 (0.65–1.51)	0.9610	0.56 (0.35–0.90)	0.0174
Extrathyroidal extension	1.41 (0.99–1.98)	0.0512	2.30 (1.67–3.16)	<0.0001
T status				
T1	Reference		Reference	
T2	1.05 (0.72–1.53)	0.8043	0.68 (0.47–0.98)	0.0377
T3	3.50 (2.37–5.17)	<0.0001	1.41 (0.95–2.07)	0.0855
T4	6.95 (3.59–13.45)	<0.0001	4.31 (2.36–7.86)	<0.0001
N status				
N0	Reference		Reference	
Nx	0.65 (0.31–1.39)	0.2655	0.66 (0.37–1.19)	0.1679
N1a	7.51 (4.75–11.86)	<0.0001	2.47 (1.54–3.97)	0.0002
N1b	9.37 (6.26–14.01)	<0.0001	4.65 (3.26–6.63)	<0.0001

OR—odds ratio, CI—confidence interval.

**Table 5:** Multivariate analysis of risk factors for predicting cancer-specific survival.

	Cancer-Specific Survival	
Risk Factor	HR (95% CI)	*p* Value
Male sex	0.96 (0.40–2.11)	0.9125
Age (>55 years)	9.96 (4.55–23.51)	<0.0001
Bilateral tumors	0.70 (0.24–2.58)	0.5630
Multifocal tumors	1.84 (0.50–5.54)	0.3309
Extrathyroidal extension	1.32 (0.52–3.52)	0.5656
T status		
T1	Reference	
T2	1.10 (0.36–3.06)	0.8655
T3	2.10 (0.78–5.51)	0.1388
T4	0.66 (0.20–1.94)	0.4630
N status		
N0	Reference	
Nx	0.78 (0.12–3.16)	0.7556
N1a	4.53 (0.96–33.72)	0.0562
N1b	1.38 (0.57–3.18)	0.4590
Type of surgery (total thyroidectomy)	2.78 (0.78–13.72)	0.1222
Radioactive iodine therapy (given)	0.39 (0.13–1.53)	0.1643
Recurrent disease (vs. persistent disease)	6.44 (2.40–22.56)	<0.0001

HR—hazard ratio; CI—confidence interval.

## Data Availability

The data presented in this study are available in the article.

## Supplementary Materials

The following supporting information can be downloaded at: https://www.mdpi.com/article/10.3390/jcm13071877/s1, Figure S1: Flowchart showing the initial treatments and outcomes for each stage of DTC. Figure S2: Flowchart showing the outcomes after the initial treatment and additional treatments that were performed after recurrent/persistent disease were established.
