# Supplementary material for: Recurrent Middle Eastern Differentiated Thyroid Carcinoma Has Worse Outcomes Than Persistent Disease
Source: J Clin Med. 2024 Mar 25;13(7):1877. doi: 10.3390/jcm13071877 (PMC11012810; doi:10.3390/jcm13071877)
Supplement: Supplementary file 1 [file jcm-13-01877-s001.zip › jcm-2882110-supplementary.pdf]

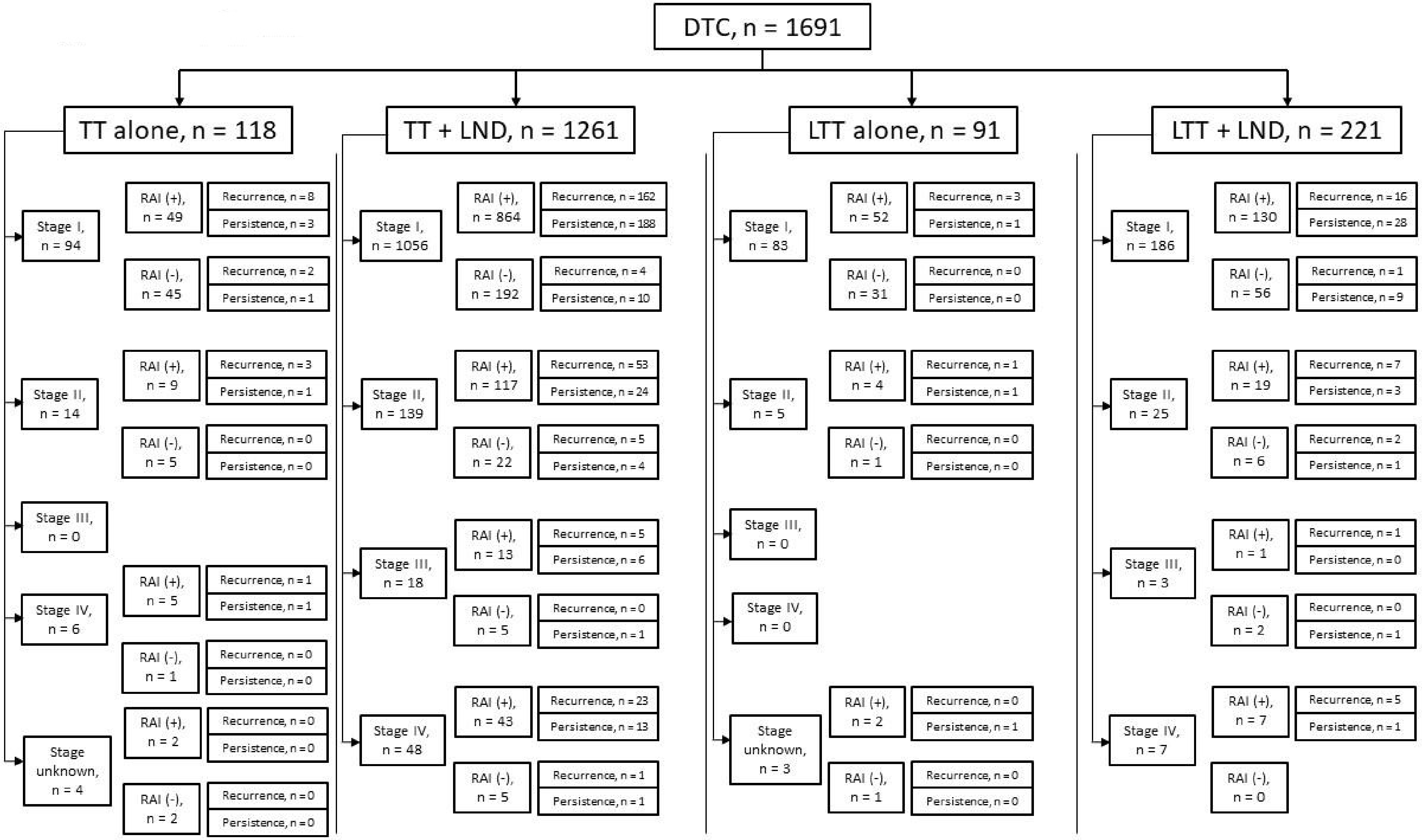

Supplementary Figure S1: Flowchart showing the initial treatments and outcomes for each stage of DTC.

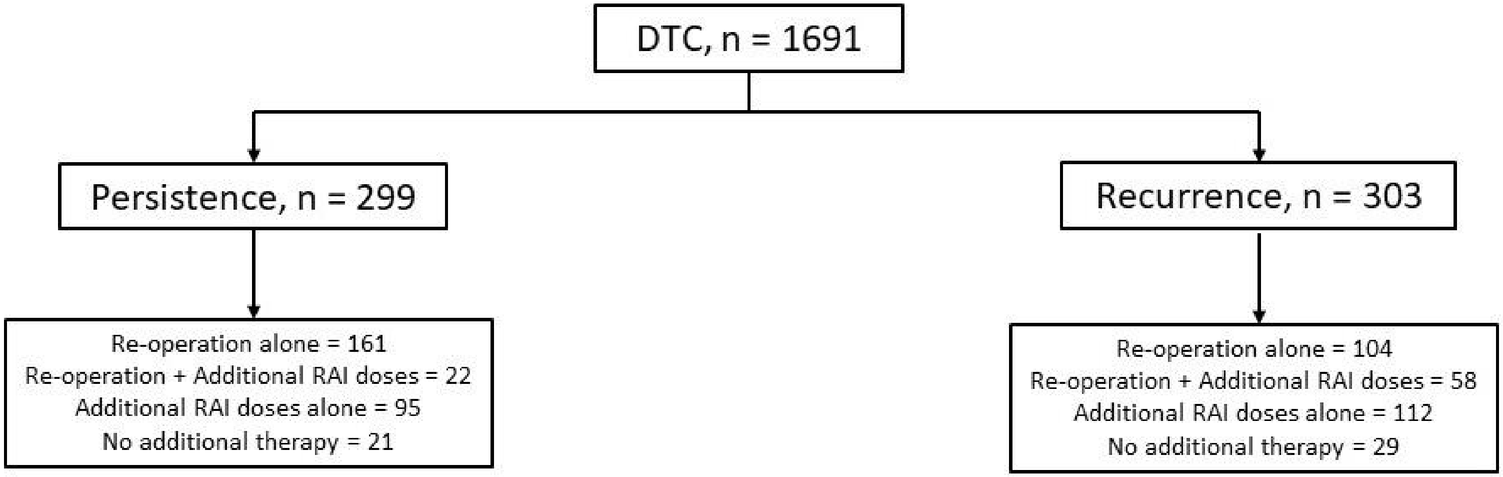

Supplementary Figure S2: Flowchart showing the outcomes after the initial treatment and additional treatments that were performed after recurrent/persistent disease were established.
